# Changing trends in the disease burden of esophageal cancer in China from 1990 to 2017 and its predicted level in 25 years

**DOI:** 10.1002/cam4.3775

**Published:** 2021-02-14

**Authors:** Songbo Li, Hui Chen, Jinyu Man, Tongchao Zhang, Xiaolin Yin, Qiufeng He, Xiaorong Yang, Ming Lu

**Affiliations:** ^1^ Clinical Epidemiology Unit Qilu Hospital of Shandong University Jinan China; ^2^ Department of Gastroenterology Qilu Hospital Cheeloo College of Medicine Shandong University Jinan China; ^3^ Clinical Research Center of Shandong University Qilu Hospital Cheeloo College of Medicine Shandong University Jinan China; ^4^ Department of Epidemiology and Health Statistics School of Public Health Cheeloo College of Medicine Shandong University Jinan China

**Keywords:** disease burden, esophageal cancer, prediction, risk factors, trend

## Abstract

**Background:**

Nearly half of the cases of esophageal cancer in the world were in China, but the corresponding burden in China has not been estimated for the past decades or for the near future.

**Methods:**

Data on the incidence, mortality, and disability‐adjusted life years (DALYs) rates owing to esophageal cancer in China from 1990 to 2017 were extracted from the Global Burden of Disease Study 2017. To reflect the trend in the disease burden, we calculated the estimated annual percentage change (EAPC) in the age‐standardized rates of these three outcomes in China from 1990 to 2017.

**Results:**

The age‐standardized incidence rate (ASIR) for esophageal cancer decreased from 19.38/100,000 in 1990 to 12.23/100,000 in 2017, with an EAPC of −2.53 (95%CI: −2.90, −2.16), but the number of cases of esophageal cancer increased from 164,473 to 234,624. The age‐standardized rates of esophageal cancer in females were always lower than they were in males during the study period, and there was a downward trend that was more pronounced among females than males. The most common risk factors for males were smoking and alcohol consumption, while the most common risk factors for females were a diet low in fruits and a high body mass index (BMI). New cases of, and deaths from esophageal cancer are predicted to increase by about 1.5 times in the coming 25 years.

**Conclusion:**

Although the age‐standardized burden of esophageal cancer has been declining, the number of new cases of, and deaths from esophageal cancer have increased in China over the past 30 years, and they will continue to increase in the near future. Hence, national policies should be adopted to promote the prevention and management of known risk factors for it, especially smoking and excessive caloric intake.

## Graphical abstract

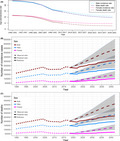

## 1 INTRODUCTION

Esophageal cancer is one of the major reasons for the global burden of cancer,^1^, ^2^, ^3^ with 473,000 new cases and 436,000 deaths in 2017, worldwide. Esophageal cancer is classified as squamous cell carcinoma (SCC) and an adenocarcinoma (AC) histologically. About 88% of global cases of esophageal cancer are SCC, especially in South‐eastern and Central Asia.^4^ Nearly half of the new cases of esophageal cancer in 2017 were in China due to its high incidence rate and China's large population (234,624 Chinese cases of the 472,525 global incidence of cases: 49.7%). Similar to global trends, China had a reduction in the age‐standardized incidence, mortality, and disability‐adjusted life years (DALYs) rates of esophageal cancer from 1990 to 2017. Nevertheless, the total number of new cases during this period grew by 42.7%, from 164,000 to 235,000.^3^

The prevention of esophageal cancer has been an important goal of the Chinese government. Since the 1960 s and 1970 s, Chinese scientists have conducted a large number of epidemiological studies in many areas of the country with a high incidence of esophageal cancer, such as Linzhou and Cixian. Nitrosamines, lack of nutrients, and unhealthy lifestyles have been identified as major risk factors for esophageal cancer. Accordingly, preventive measures have been implemented to combat the prevalence of esophageal cancer, such as improving toilet sanitation, increasing nutritional supplements, and changing unhealthy lifestyles.^5^ Over the past 30 years, with urbanization, aging, and rising incomes, identifying various other risk factors for esophageal cancer has been difficult, posing a massive challenge for China's public health system. Previous studies on the prevention of esophageal cancer have included only single‐centers, used small samples, and lacked continuity and unified standards, which limit the value of their findings. Rarely have studies in China performed specific analyses of trends in esophageal cancer over time in terms of its incidence, mortality, and DALYs rates in relation to the major risk factors of sex and age. Therefore, it is urgent to understand the epidemic trend of esophageal cancer in China in order to establish relevant health policies that can guide practices to prevent and manage esophageal cancer.

The Global Burden of Disease (GBD) database of the Institute of Health Measurement and Evaluation (IHME) consists of a large sample of panel data on the incidence, death, and DALYs rates of esophageal cancer from 1990 to 2017. The analysis presented in this study focused on secular trends over 25 years in the disease burden of esophageal cancer by sex, age, and other risk factors in China. Our aim was to provide an evidence‐based assessment of the efficacy of current prevention and therapeutic strategies, in order to reduce the burden of esophageal cancer in China.

## 2 MATERIALS AND METHODS

### 2.1 Data sources

Data on the incidence, mortality, and DALYs rates of esophageal cancer in China from 1990 to 2017 were extracted from the official website of the GBD 2017 Study,^6^ which is available from the Institute for Health Metrics and Evaluation (IHME) for free (http://ghdx.healthdata.org/gbd‐results‐tool). The GBD annually provides age‐sex‐specific incidence, death, and DALYs rates for countries throughout the world. Previous studies have described the detailed methodology of the GBD 2017 Study.^7^, ^8^ We chose “China” from the database as the location, “esophageal cancer” for the cause, and “death,” “incidence,” and “disability‐adjusted life‐years (DALYs)” for measures. In this study, we present the incidence, mortality, DALYs rates for esophageal cancer in China by sex and age. The study also presents the percentage change of these indicators from 1990 to 2017 to reflect the trends in cancer burden. The estimated population of China was taken from the United Nations World Population Prospects 2019 Revision, by year (up to 2100), age, and sex (https://population.un.org/wpp/Download/Standard/Population/).

### 2.2 Statistical analysis

Descriptive analyses were conducted on esophageal cancer deaths, incidence, and DALYs data by gender, age, and year. Cases were divided into 5‐year age‐groups to describe the age‐related incidence, mortality, and DALYs rates of esophageal cancer in 2017. To reflect the trends in cancer burden, we also calculated the estimated annual percentage change (EAPC) in the age‐standardized incidence rate (ASIR) and the age‐standardized mortality rate (ASMR) for esophageal cancer nationwide from 1990 to 2017. The calculation was based on a regression model fitted to the natural logarithm of the rate, namely ln(rate) = α + β*(calendar year) + ε. EAPC was defined as 100 × (exp(β)–1); the 95% confidence interval (CI) of the EAPC was also determined by the fitted model. The changes in each outcome were calculated by comparing the data for the year 2017 to the data for the year 1990. All data analyses were conducted using the open‐source software R (version 3.6.2); the packages included ggplot2 and RColorBrewer. Moreover, we predicted the number of new cases and deaths due to esophageal cancer from 2017 to 2042 by running an Nordpred age‐period‐cohort (APC) analysis by sex using the Nordpred package in R, taking into account the changing rates and changing population structure, which has been fully demonstrated and recognized in previous studies.^9^ In addition, in order to facilitate comparison with the predicted results, we calculated the absolute number of events that would occur if the rates remained stable (baseline reference), decreased by 1% per year (optimistic reference), and increased by 1% per year (pessimistic reference), based on the actual observed rates in 2017. To validate the stability of the prediction results, the Bayesian APC model integrated nested Laplace approximations (INLA) was further applied to perform a sensitivity analysis using the BAPC and INLA packages in R.^10^

## 3 RESULTS

### 3.1 Current esophageal cancer burden in China

At the national level, China had the largest number of new cases (234,624 [95%UI: 223,240–246,036]), deaths (95%UI: 212,586 [202,673–222,654]), and DALYs (95%UI: 446,4980 [4,247,816–4,690,846]) rates in 2017, accounting for almost half of the worldwide cases in that year. The ASIR was 12.23/100,000 (95%UI: 11.64, 12.82), the ASMR was 11.25/100,000 (95%UI: 10.73, 11.77), and the age‐standardized DALYs rate of esophageal cancer was 222.58/100,000 (95%UI: 211.95, 233.57) in China in 2017 (Table S1).

**FIGURE 1:**
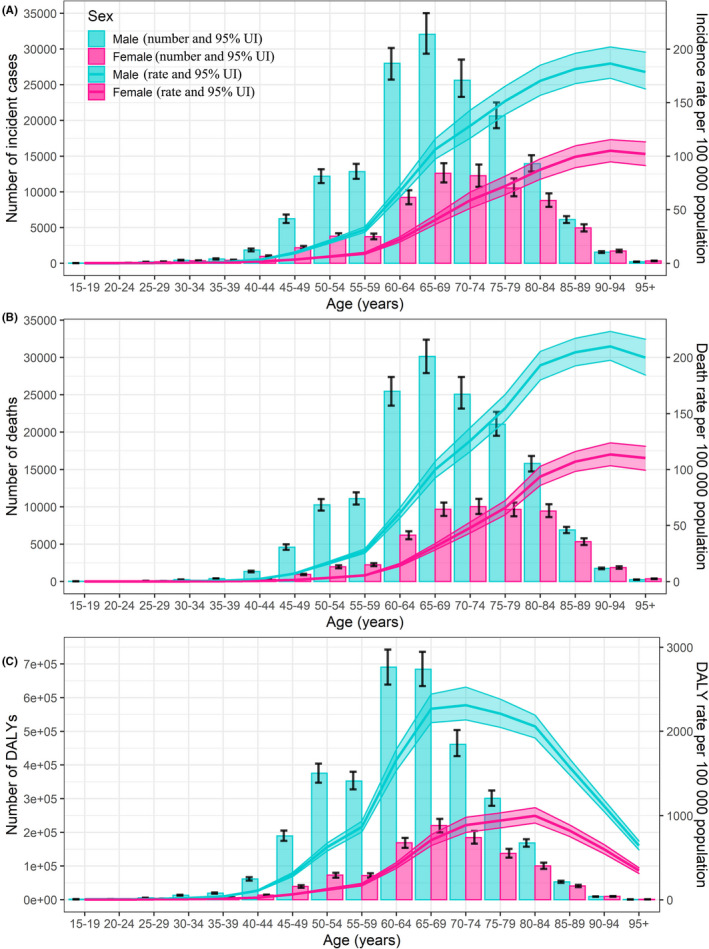
The national numbers and age‐standardized rates of incidence (A), mortality (B), and DALYs (C) of esophageal cancer per 100,000 population by age and sex, 2017 Shading indicates the upper and lower limits of the 95% uncertainty intervals (95% UIs). DALYs, disability‐adjusted life‐year

Figure 1 presents the incidence, mortality, and DALYs number and rate of esophageal cancer in China by gender and age in 2017. The ASIR of males was 2.4 times higher, the ASMR rate was 2.8 times higher, and the DALYs rate was 3.2 times higher than those of females. Because esophageal cancer is a deadly disease with high mortality, its incidence and mortality have similar characteristics and trends. From Tables S1–S3, it can be seen that the incidence and death rates of esophageal cancer were less than 10/100,000, and the DALYs rate was less than 200/100,000 in patients younger than 50 years old. However, once patients reached the age of 60 years, these rates rose rapidly. The incidence and death rates of esophageal cancer surged to about 40/100,000, and the DALYs rate increased to about 1,000/100,000. We found the highest esophageal cancer incidence, death, and DALYs rates among patients 60–74 years old. The incidence and death rates increased with age increasing, reaching 132.54/100,000 and 146.46/100,000, respectively, in the 85–89‐year‐old group, but the DALYs rates peaked around 75 years of age, at a rate of 1,583.9/100,000. (95% UI: 1,487.3, 1,695.4), especially in males (Figure 1C).

### 3.2 Trends in esophageal cancer incidence, mortality, and DALYs rates over time

The ASIR of esophageal cancer decreased from 19.38/100,000 (95%UI: 18.52–20.50) in 1990 to 12.23/100,000 (95%UI: 11.64, 12.82) in 2017, with an EAPC of −2.53 (95%CI: −2.90, −2.16) (Table S2). The ASMR of esophageal cancer decreased significantly from 20.53/100,000 (95%CI: 19.60–21.71) in 1990 to 11.25/100,000 (95%CI: 10.73–11.77) in 2017, with an EAPC of −2.59 (Table S3). The standardized DALYs rate of esophageal cancer significantly decreased from 446.42/100,000 (95%CI: 425.73, 471.70) in 1990 to 222.58/100,000 (95%CI: 211.95, 233.57) in 2017, with an EAPC of −3.00 (95%CI: −3.36, −2.65) (Table S4).

**FIGURE 2:**
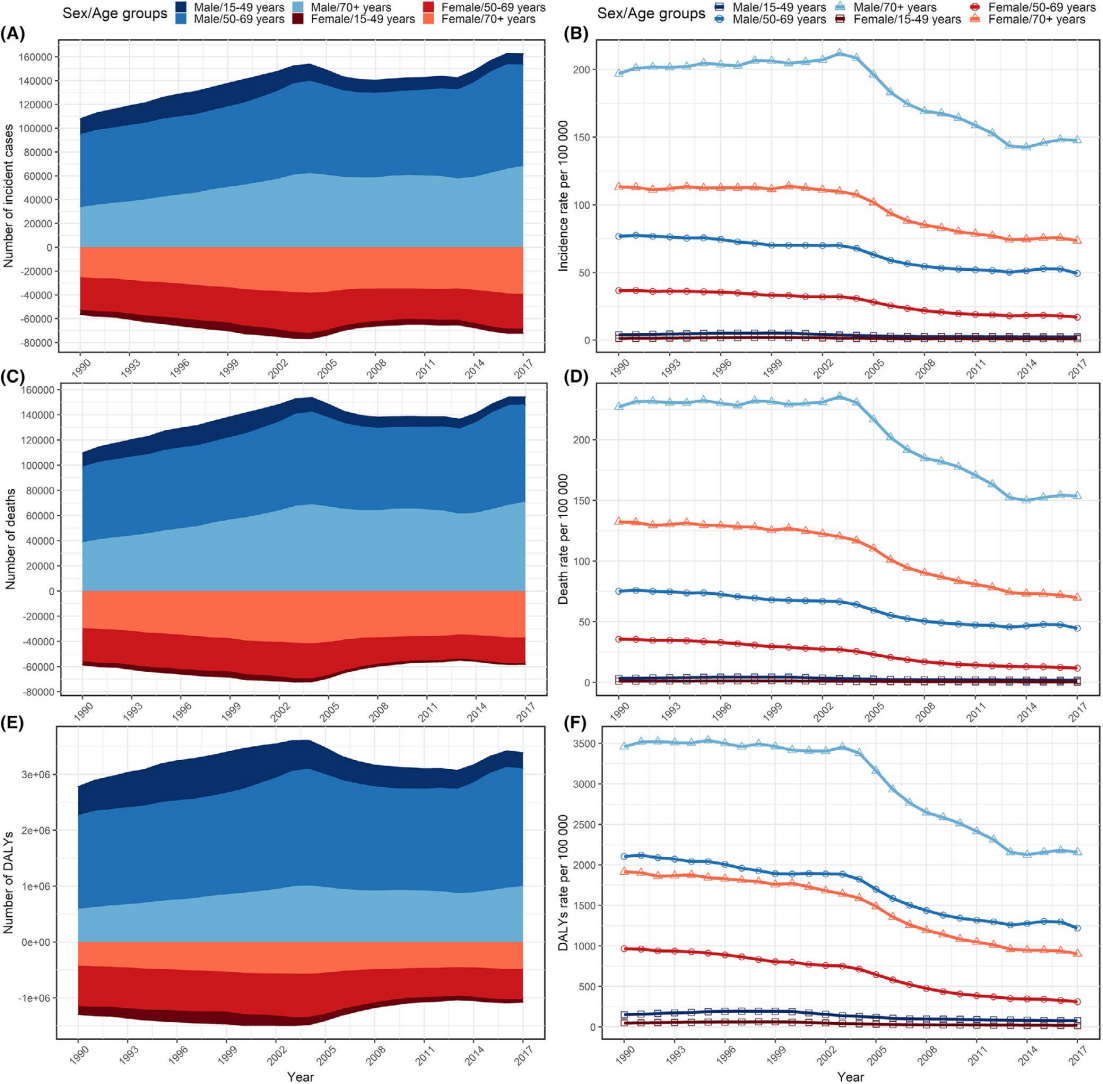
The incident number (A), incidence rate (B), deaths number (C), death rate (D), DALYs number (E), and DALYs rate (F) of esophageal cancer per 100,000 population by sex in different age groups, from 1990 to 2017. DALYs, disability‐adjusted life‐year

We found the decreased EAPCs of esophageal cancer burden in younger population was pronounced, and showed a positive linear relationship with age increasing (Figure [Link], [Link]). Simply, we analyzed temporal trends based on the gender of patients in three different age groups: 15–49 years, 50–69 years, and above 70 years. The results showed that the incidence, mortality, and DALYs rates for esophageal cancer among males were higher in 2017 compared to females in all age groups. Significant decreases in incidence, mortality, and DALYs rates were found in both genders (Figure 2, Table S2–S4). However, females showed a much more obvious decline in the ASMR (by 57.0% [52.0 to 62.0]) than males did (by 38.8% [33.4–43.9]) from 1990 to 2017 in China (Figure 2C).

The apparent discordance between younger and older patients regarding esophageal cancer‐related incidence, death, and DALYs rates prompted us to do an age‐stratified analysis of trends. The incidence, mortality, and DALYs rates of esophageal cancer in the groups younger than 50 years old were significantly lower than those who were 50 years or older from 1990 to 2017 (Figure 2). However, the EAPCs of the incidence, mortality, and DALYs rates from 1990 to 2017 had more pronounced changes in the groups under 50 years old (Tables S2‐S4).

### 3.3 Risk factors for esophageal cancer

**FIGURE 3:**
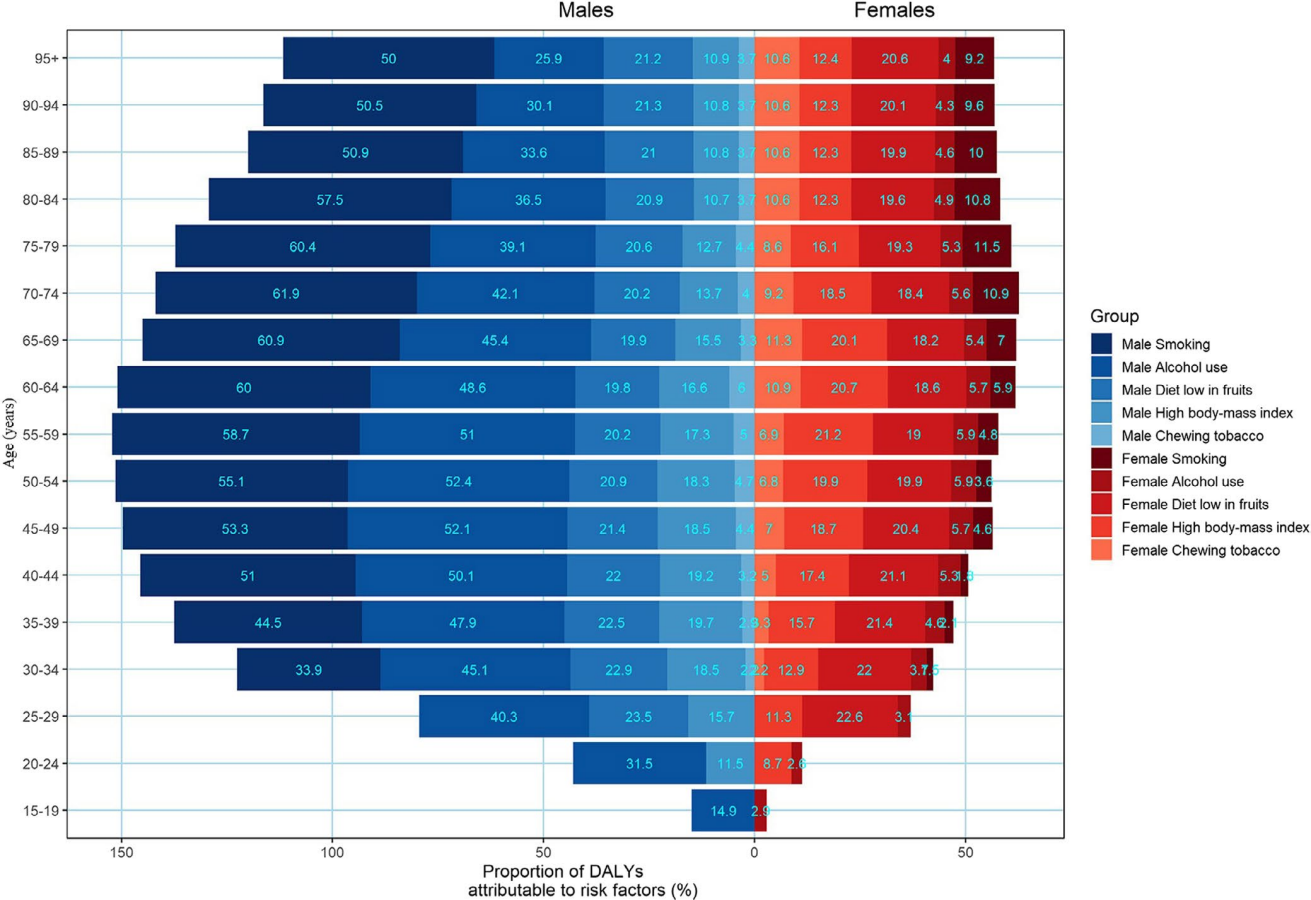
Proportions of DALYs attributable to risk factors by age and sex in 2017

At the national level, a considerable proportion of the DALYs rate was attributable to the five risk factors available from the GBD 2017 Study, including 45.9% (95%UI: 42.3–49.7) attributable to tobacco smoking, 35.6% (95%UI: 28.1–43.2) attributable to alcohol consumption, 16.0% (95%UI: 4.9–32.7) attributable to a high BMI, 20.0% (95%UI: 4.4–36.2) attributable to a diet low in fruits, and 5.6% (95%UI: 3.6–7.9) attributable to the use of chewing tobacco. Due to differences in lifestyles, the risk factors to which the genders and the various age groups were exposed varied widely. The most common risk factors for males were smoking and alcohol consumption, while the most common risk factors for females were a diet low in fruits and a high BMI. A mild reduction in esophageal cancer due to alcohol consumption was found among males older than 50 years of age. However, no significant trend was observed in different age groups with esophageal cancer due to smoking in males (Figure 3).

**FIGURE 4:**
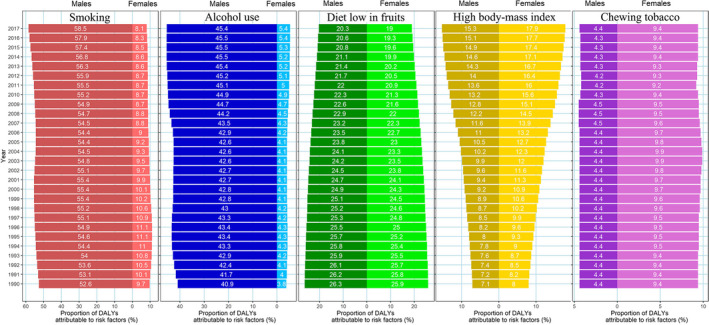
Proportions of esophageal cancer due to specific risk factors in China from 1990 to 2017

In this study, we fully analyzed the trends in China of DALYs rates due to esophageal cancer caused by distinct risk factors. No effective reduction was observed in the DALYs rates of esophageal cancer due to smoking, alcohol use, or chewing tobacco from 1990 to 2017 (Figure 4A, 4B, and 4E), while a minor increase in rate was observed in patients with esophageal cancer due to smoking from 2006 (95%UI: 54.4%, 49.4–59.2) to 2017 (95%UI: 58.5%, 54.0–63.0) among males (Figure 4A). Somehow, the proportion of esophageal cancer due to chewing tobacco was higher among females than it was among males, and the proportions were almost the same every year from 1990 to 2017 (Figure 4E). Among the risk factors investigated in this study, the DALYs rate of esophageal cancer due to a diet low in fruits and a high BMI showed the most pronounced changes (Figure 4C and 4D). The DALYs rate of esophageal cancer due to a diet low in fruits showed a small decrease from 26.3% in 1990 to 20.3% in 2017 among males, and 25.9% in 1990 to 19.0% in 2017 among females (Figure 4C). The proportion of esophageal cancer due to a high BMI showed the most pronounced increase, especially among females (from 8.0% in 1990 to 17.6% in 2017) (Figure 4D).

**FIGURE 5:**
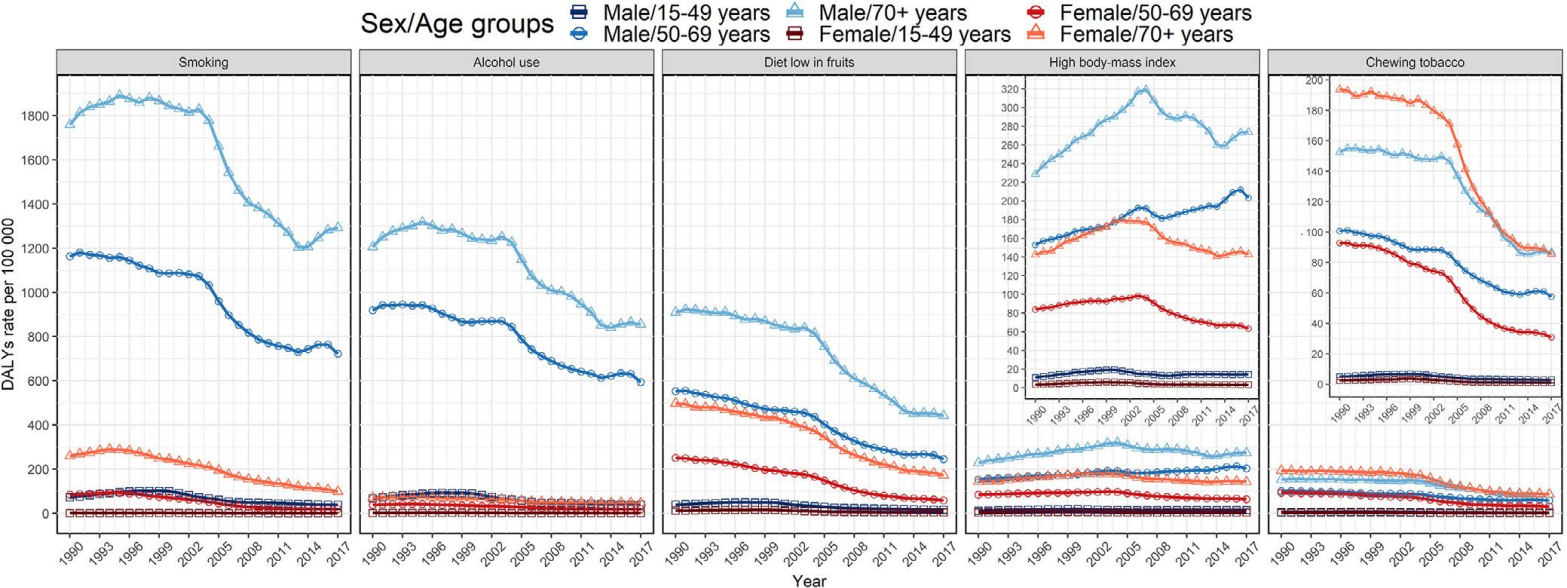
DALY rates of esophageal cancer due to specific risk factors by sex in different age groups in China from 1990 to 2017

In addition, we found distinctive characteristics of these risk factors in different age groups. Almost all the risk factors for esophageal cancer had much higher DALYs rates among people over 50 years old, especially for males. The DALYs rates due to most risk factors showed an obvious downward trend for patients from 1990 to 2017, except for the high BMI. The DALYs rate due to high BMI even increased in most age groups among males from 1990 to 2017, but the females showed an opposite trend in the age group of 50–69 years (Figure 5).

### 3.4 Predictions of esophageal cancer incidence and death rates in China

**FIGURE 6:**
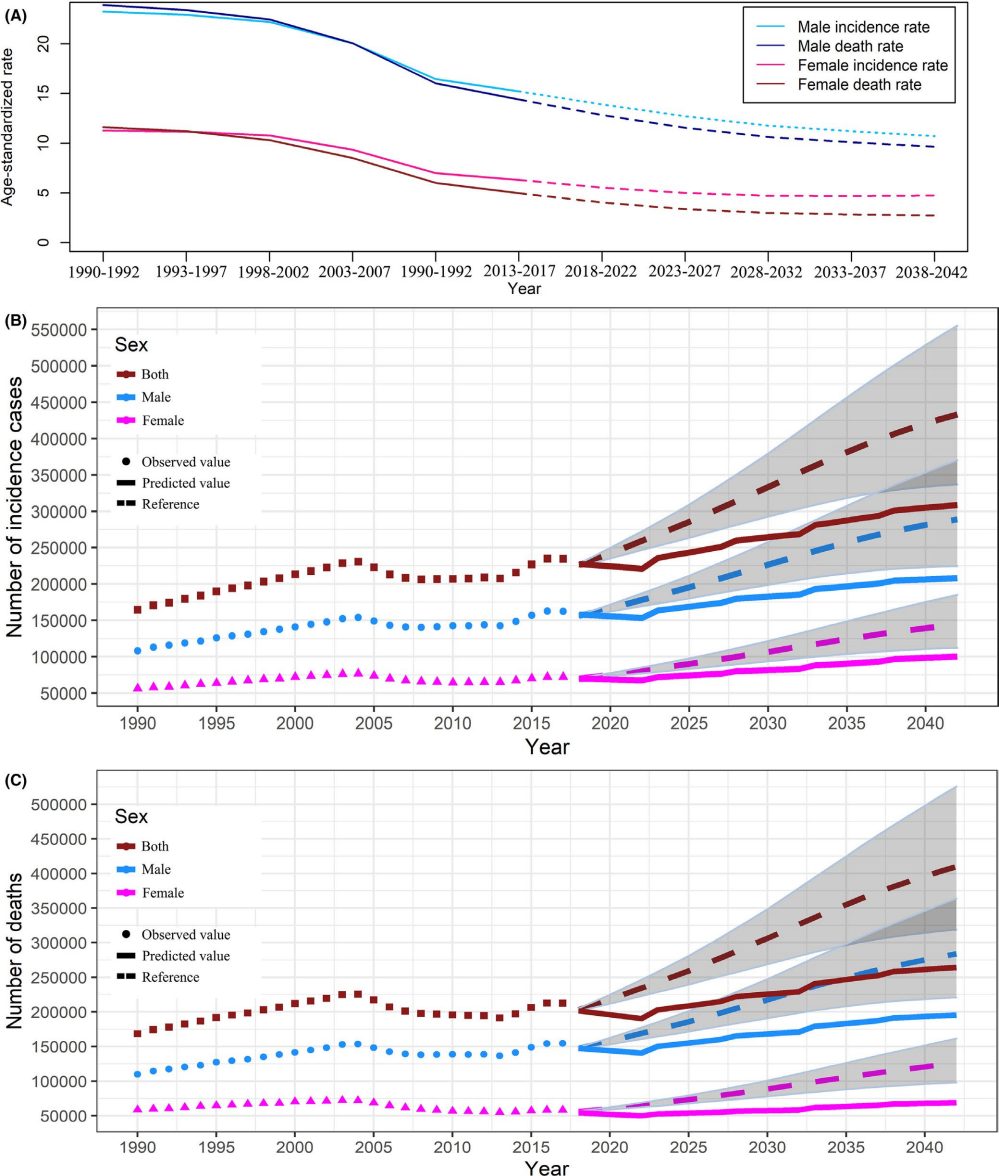
Trends in esophageal cancer incidence and death rates by sex in China: observed (solid lines) and predicted rates (dashed lines) (A). Trends in observed (dashed lines) and predicted (solid lines) esophageal cancer in number of incidence cases (B) and deaths(C). Shading indicates if the rate remained stable (baseline reference), decreased by 1% per year (optimistic reference, lower limit), and increased by 1% per year (pessimistic reference, upper limit) based on the observed rate in 2017

We predicted the future incidence and death rates of esophageal cancer would continue to decrease in China, especially among males. Although the trends of incidence and death rates in females were somewhat similar to those in males, incidence and death rates were substantially lower than in males and the death rates showed a more pronounced decline than the incidence. Furthermore, the incidence and death rates were predicted to relatively stabilize after 2030, and the death rate was predicted to remain at a relatively low level, largely due to progress in a series of therapeutic progress (Figure 6A).

Despite the estimated decreases in the incidence of esophageal cancer, the numbers of new cases and deaths would be expected to continue to increase in China during the next 25 years due to population growth and aging. Shading in Figure 6 indicated a huge variation in the number of new cases and deaths if the corresponding rate increases or decreases by 1% annually, which further highlighted the importance of prevention and treatment of esophageal cancer. Although our predicted results were somewhat lower than the optimistic reference with 1% declined rate annually, the number of new cases would increase from 162,500 in 2017 to 208,300 in 2042 among males, and the number of deaths would increase from 154,400 to 195,500 during the same period. Among females, the number of new cases would increase from 72,200 in 2017 to 100,400 in 2042. Consequently, the number of deaths due to esophageal cancer would increase from 58,200 to 68,800 (Figure 6B and 6C).

In order to assess the robustness of our predictive results, we further performed a sensitivity analysis using the Bayesian APC model to predict the future burden of esophageal cancer in China. The results of the Bayesian APC model showed consistent trends with the above‐mentioned results, except for the last 5 years (Figure [Link], [Link]). For simplifying the description of the predicted results, the results of the sensitivity analysis were provided as supplementary materials to show the stability of the prediction results.

## 4 DISCUSSION

China is one of the countries with the highest incidence of esophageal cancer in the world, and the DALYs rate due to esophageal cancer in China ranks 4th among the rate for all cancers.^11^ Although the ASIR and ASMR of esophageal cancer have decreased in China in the last 30 years, the total numbers of new cases of, and deaths caused by esophageal cancer have increased because of changes in the population's age and structure. The trend in the incidence due to esophageal cancer in some high‐incidence areas in China is similar to the results of this study. For example, the ASIR was 125.24/100,000 in 1988 and 81.78/100,000 in 2003 in Linzhou City, a decrease of 34.7%.^12^ However, differences in specific pathological patterns, regions, sexes, and age groups have resulted in a markedly diverse esophageal cancer burden in China, making the prevention and control of esophageal cancer complex. These temporal trends show that esophageal cancer remains a major disease burden in China, and its total burden may continue to increase.

The incidence of esophageal cancer is low during youth, and increases with age, reaching a peak among 70‐ and 80‐year‐olds, largely due to longer exposure to those risk factors mentioned above and decreases in physiological function and immunosurveillance. Moreover, most risk factors showed an obvious decrease in young people in the current study, and a pronounced decrease in the incidence and mortality among the same groups was also observed from 1990 to 2017, which demonstrates that effective interventions can further reduce the incidence of esophageal cancer, especially in men. In addition, esophageal cancer is more common in men, and it incidence rate is four to six times higher in males than in females.^13^, ^14^

Previous studies on the attributable risk of esophageal cancer in China showed 46% of esophageal cancers (51% in men and 33% in women) were attributable to tobacco smoking, alcohol drinking, low vegetable intake, and low fruit intake.^15^ Tobacco smoking and alcohol use are the main risk factors for SCC. ^16^, ^17^, ^18^ Together, they are associated with over 75% of cases of SCC in developed countries.^19^, ^20^ The risk of AC is about twice as high in current smokers as it is in people who have never smoked, but tobacco smoking is a more serious risk factor for SCC than AC.^21^, ^22^ Unlike AC, the incidence of SCC among people who drink alcohol three or more drinks a day is probably three to five times higher, and the risk increases in conjunction with smoking. Some studies have demonstrated that the risk of esophageal cancer decreases substantially after smoking cessation.^23^ However, the current study found no significant reduction in the DALYs rate of esophageal cancer caused by smoking or alcohol use from 1990 to 2017. Smoking is still a serious public health problem in China, although tobacco control policies have been implemented since the signing of the World Health Organization Framework Convention on Tobacco Control in 2003.^24^ More importantly, alcohol misuse among teenagers is an emerging problem in China.^25^ Therefore, policies aimed at preventing further growth in alcohol consumption should be introduced, such as strong restrictions on the sale and consumption of alcoholic beverages.

Obesity or a high BMI has been identified as the strongest risk factor for AC.^17^, ^26^, ^27^ Previous studies on the global or national cancer burden have assessed the combined effects of total vegetable and fruit intake.^17^, ^28^, ^29^ Excessive intake of processed foods and fats increases the risk of both SCC and AC, whereas a high intake of fresh fruit and dietary fiber reduces the risk.^30^, ^31^ However, the prevalence of risk factors such as smoking, obesity, and diabetes continues to rise,^32^, ^33^, ^34^ despite the fact that measures have been taken to counter these risks.

Considering the low survival rates in patients with esophageal cancer, major measures should be taken to reduce these risk factors, especially as most of these risk factors (e.g., obesity and smoking) are also serious risk factors for many common chronic diseases. Various changes in society are expected to affect the incidence and mortality of esophageal cancer, especially progress on a series of diagnostic and therapeutic measures, such as screening, endoscopic submucosal dissection, endoscopic mucosal resection, photodynamic therapy, neoadjuvant radiotherapy and chemotherapy, targeted therapy, and so on.^35^, ^36^ Randomized controlled trials have shown that screening is related to a decrease in death rate.^37^, ^38^, ^39^ The poor prognosis of esophageal cancer may also require screening efforts in high‐risk groups.

Further studies are needed to evaluate detection strategies based on the age and risk factors of individuals. We hope that the incidence of esophageal cancer can be further reduced by trying to change people's lifestyles and by reducing the screening age of gastrointestinal endoscopy for precancerous lesions of esophageal cancer to detect it as early as possible.

Limitations exist in this study in terms of the comprehensiveness and timeliness of its information. For instance, the two main histological subtypes of esophageal cancer, namely SCC and AC, have distinct risk factors and incidence trends, but currently there are no separate data for these two subtypes in the GBD database. In addition, there is increasing evidence that indoor air pollution and exposure to biomass smoke,^40^, ^41^ lack of tap water, bad oral health,^42^ and drinking very hot beverages^43^, ^44^ are risk factors for SCC; yet, the GBD 2017 Study did not have data on these variables. Therefore, special surveys should be conducted that focus on these risk factors of esophageal cancer to improve the relevant information available.

## 5 CONCLUSIONS

Our study presents evidence for reductions in ASIR and ASMR over the past 30 years. However, the number of new cases of, and deaths from esophageal cancer increased from 1990 to 2017, and will continue to increase in China as the combined result of increasing high‐risk behaviors (e.g., drinking and tobacco smoking) and changing demographics over the next decades.

The burden of esophageal cancer will increase by about 1.5 times in the next 25 years. Therefore, reinforced prevention and management of major known and potential esophageal cancer risk factors, like alcohol consumption, smoking, and poor diet, should be strongly recommended and improved through effective national policies, which might contribute to variations in the incidence of esophageal cancer. In the meantime, direct measures and strategies aimed at improving diagnosis and treatment should be proposed to curb the growth trend in the incidence of esophageal cancer and reduce the burden of esophageal cancer.

## Ethics Statement

Ethics approval was exempted by the Ethics Committee of Qilu Hospital of Shandong University, because the GBD is a publicly available database and all participants’ data were anonymous.

## CONFLICT OF INTEREST

The authors state that the study was conducted without any commercial or financial relationships, which may be interpreted as potential conflicts of interest.

## Supporting information

Figure S1

Figure S2

Figure S3

Figure S4

Tables S1‐S4

## Data Availability

All data could be extracted from the online GBD 2017 database.
