# Supplementary figures and images for: Changing trends in the disease burden of esophageal cancer in China from 1990 to 2017 and its predicted level in 25 years
Source: Cancer Med. 2021 Feb 14;10(5):1889–99. doi: 10.1002/cam4.3775 (PMC7940228; doi:10.1002/cam4.3775)

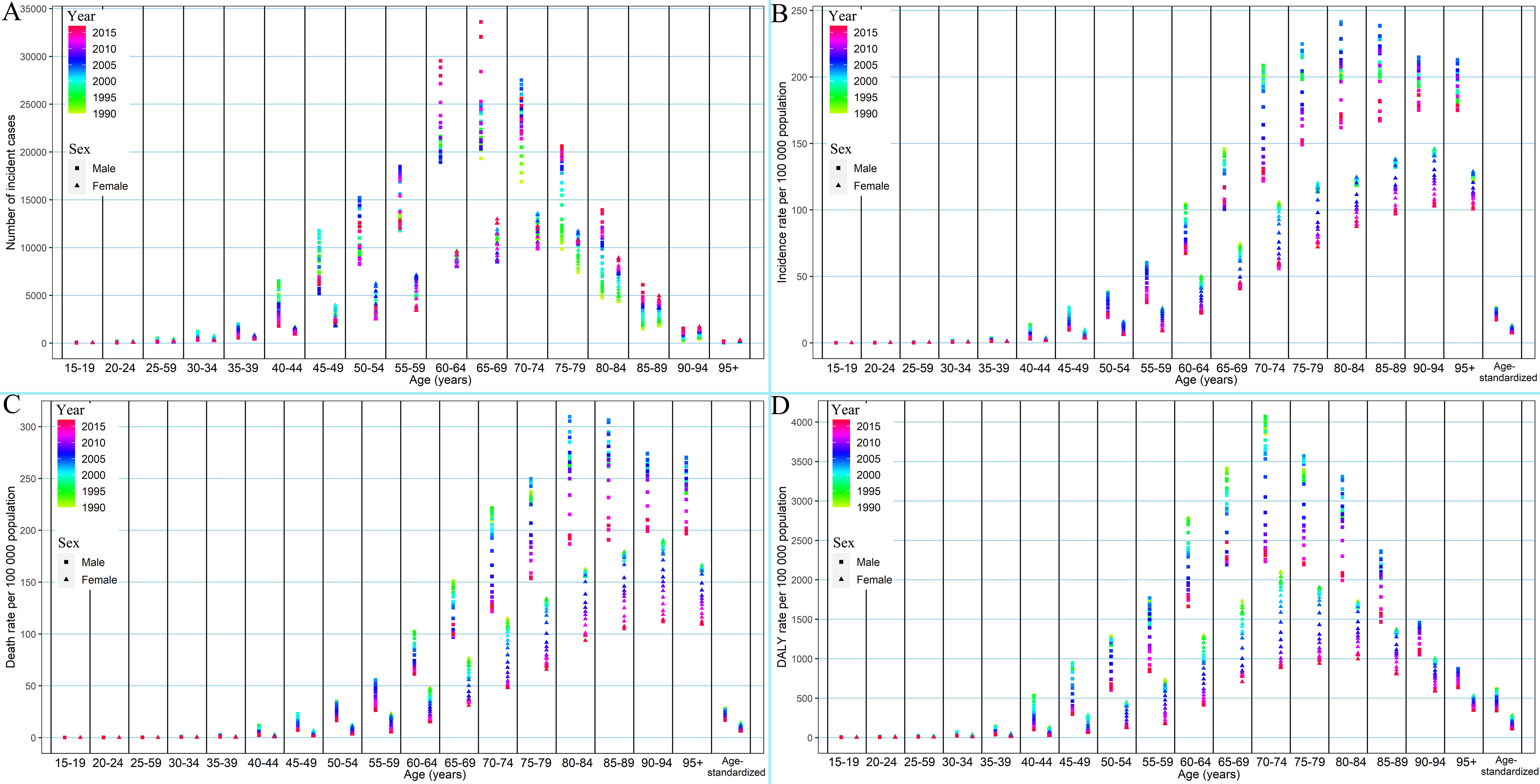

Supplement: Supplementary file 1 — Figure S1 [file CAM4-10-1889-s005.jpg]

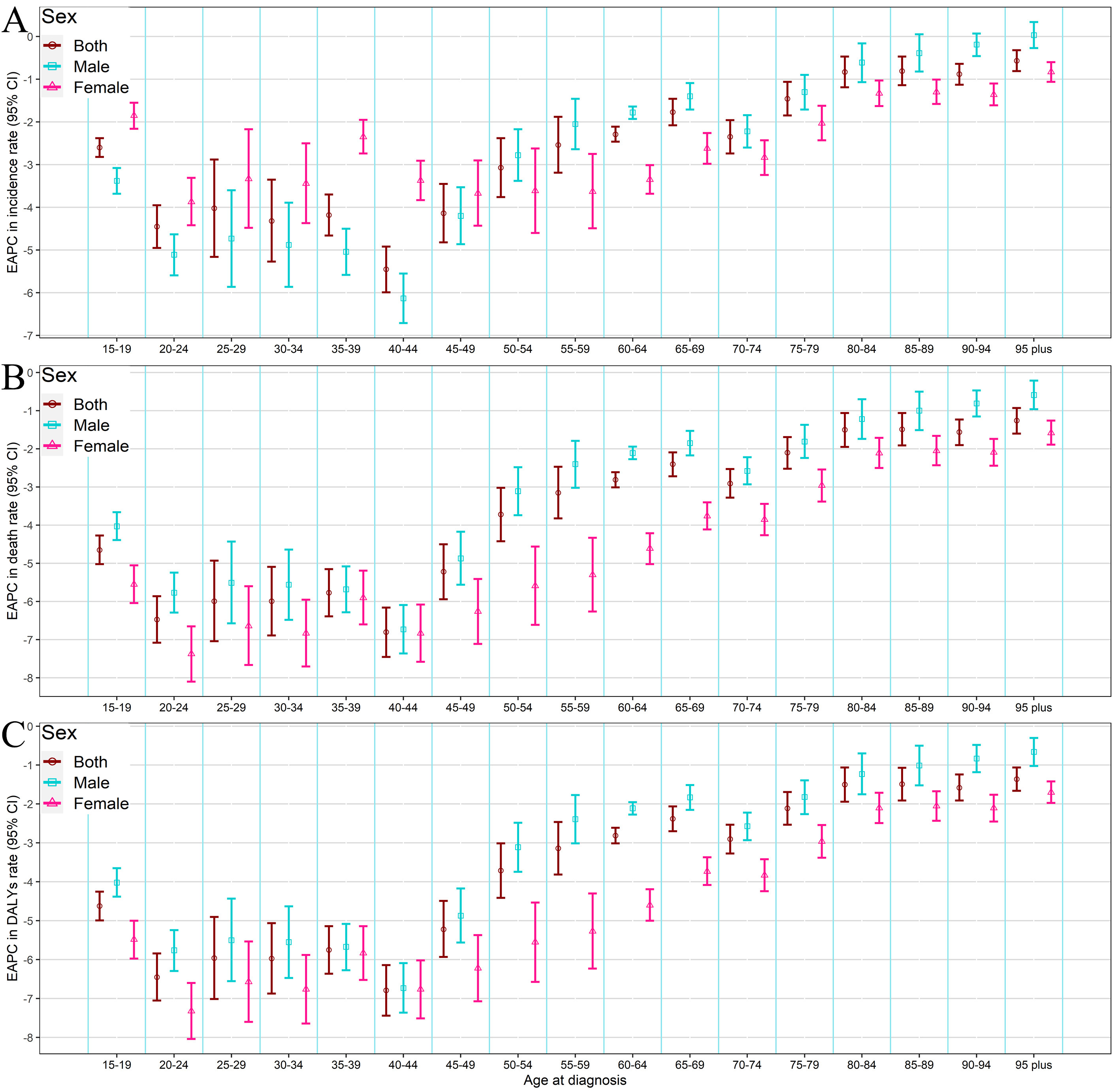

Supplement: Supplementary file 2 — Figure S2 [file CAM4-10-1889-s001.jpg]

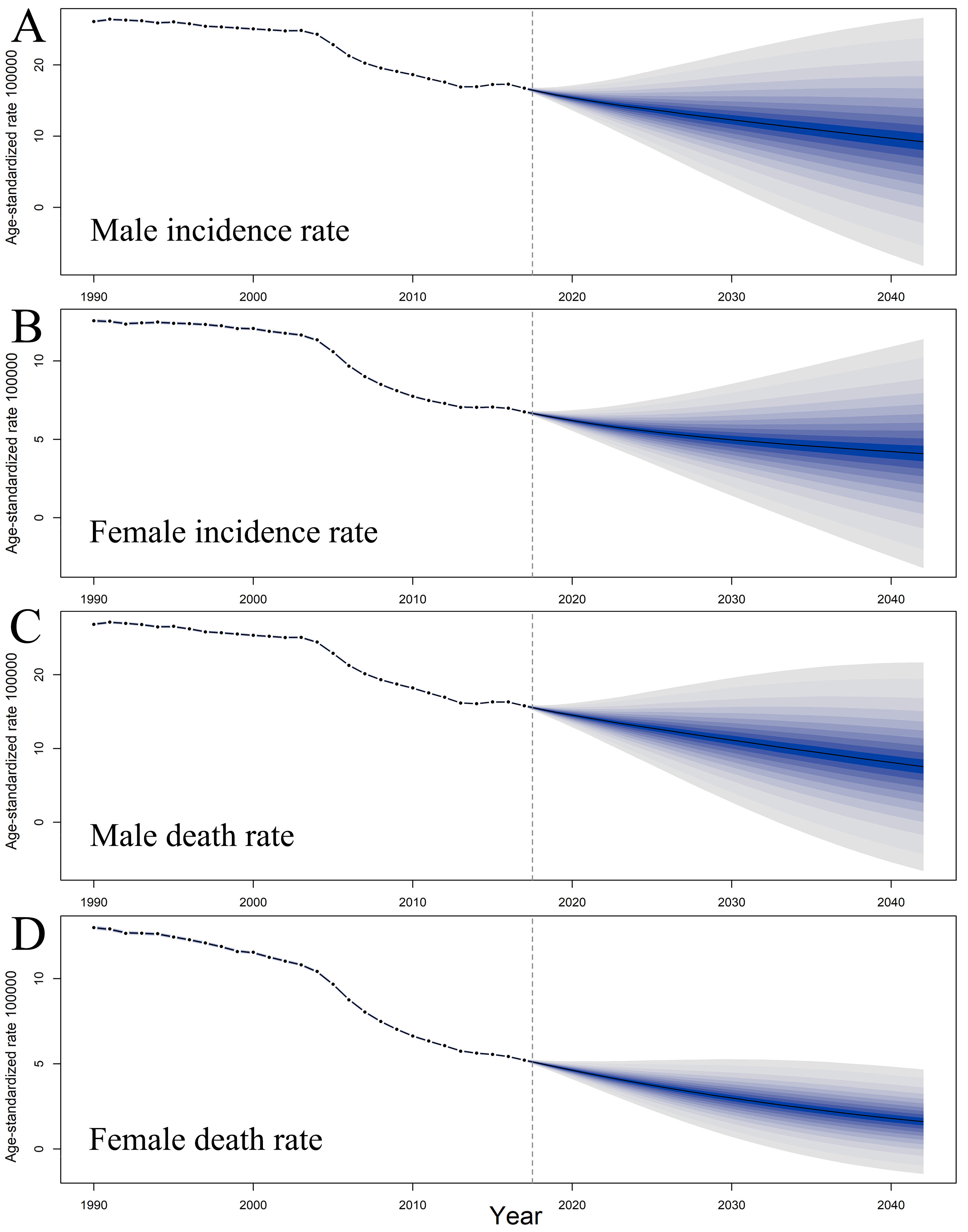

Supplement: Supplementary file 3 — Figure S3 [file CAM4-10-1889-s004.jpg]

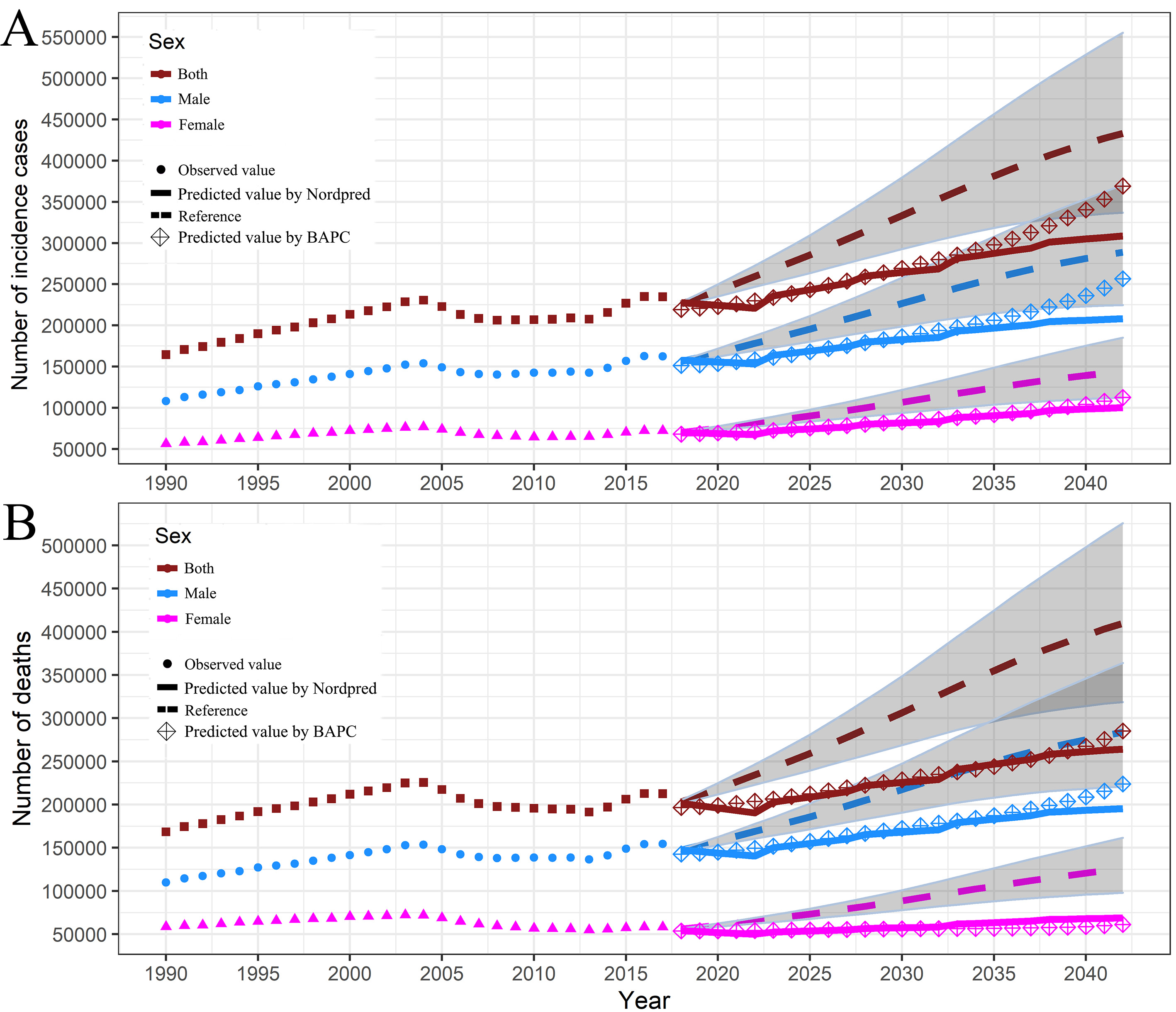

Supplement: Supplementary file 4 — Figure S4 [file CAM4-10-1889-s002.jpg]
