# Supplementary material for: Changing trends in the disease burden of esophageal cancer in China from 1990 to 2017 and its predicted level in 25 years
Source: Cancer Med. 2021 Feb 14;10(5):1889–99. doi: 10.1002/cam4.3775 (PMC7940228; doi:10.1002/cam4.3775)
Supplement: Supplementary file 5 — Tables S1‐S4 [file CAM4-10-1889-s003.doc]

**Changing Trends in the Disease Burden of Esophageal Cancer in China from 1990 to 2017 and Its Predicted Level in 25 years**

**Running title: Trends and Predictions of Esophageal Cancer**

**4 supplementary tables and 4 supplementary figures.**

**Contents**

**Table S1:** Incident cases, Death and DALYs of esophageal cancer in 1990 and 2017 for both sexes and percentage change of age-standardized rates per 100 000.

**Table S2:** The incident cases and age‐standardized incidence of esophageal cancer in 1990 and 2017 and its temporal trends from 1990 to 2017

**Table S3:** The number of deaths and age‐standardized mortality rates of esophageal cancer in 1990 and 2017 and the temporal trends from 1990 to 2017

**Table S4:** The number of DALYs and age‐standardized DALY rates of esophageal cancer in 1990 and 2017 and the temporal trends from 1990 to 2017

**Figure S1.** The number of incident cases (A), incidence rate (B), mortality rate (C), and DALY rate (D) of esophageal cancer per 100 000 population by age and sex, from 1990 to 2017. DALY=disability-adjusted life-year.

**Figure S2.** The EPAC in incidence rate (A), death rate (B) and DALYs rate (C) of esophageal cancer by sex and age from 1990 to 2017. DALYs=disability-adjusted life-year.

**Figure S3.** The temporal trends of ASIR and ASMR for esophageal cancer between 1990 and 2042 in China in males (A: ASIR; C: ASMR) and females (B: ASIR; D: ASMR). The open dots represent the observational values from GBD dataset, and the blue shadow denotes the 95% highest density interval of prediction values. The predictive mean value is shown as a black solid line. The vertical dashed line indicates where the prediction starts.

**Figure S4.** Trends in observed (dashed lines) and predicted (solid lines: predicted value by Nordpred; rhombic lines: predicted value by BAPC) esophageal cancer in number of incidence cases(A) and deaths(B). Shading indicates if the rate remained stable (baseline reference), decreased by 1% per year (optimistic reference, lower limit), and increased by 1% per year (pessimistic reference, upper limit) based on the observed rate in 2017.

**Table S1: Incident cases, Death and DALYs of esophageal cancer in 1990 and 2017 for both sexes and percentage change of age-standardized rates per 100 000**

|  |  | **1900** | | **2017** | | **Percentage change in**  **age-standardized**  **rates between**  **1990 and 2017** |
| --- | --- | --- | --- | --- | --- | --- |
|  |  | Counts (95% UI) | Rate (95% UI) | Counts (95% UI) | Rate (95% UI) |  |
| **Incident cases** | Global | 310236  (300690, 322028) | 7.6  (7.3, 7.9) | 472525  (459485, 485294) | 5.9  (5.7, 6.1) | -22  (-25.2, -18.6) |
|  | China | 164473  (157194, 173853) | 19.4  (18.5, 20.5) | 234624  (223240, 246036) | 12.2  (11.6, 12.8) | -36.9  (-41.7, -32.1) |
| **Death** | Global | 311289  (301451, 323235) | 7.7  (7.5, 8) | 435959  (424994, 447580) | 5.5  (5.3, 5.6) | -29  (-32, -25.8) |
|  | China | 168455  (160933, 178231) | 20.5  (19.6, 21.7) | 212586  (202673, 222654) | 11.2  (10.7, 11.8) | -45.2  (-49.6, -41.1) |
| **DALYs** | Global | 7677078  (7422100, 7970949) | 179.9  (174.1, 186.8) | 9777771  (9532791, 10031497) | 119.9  (116.9, 123) | -33.4  (-36.1, -30.4) |
|  | China | 4078281  (3890143, 4314430) | 446.4  (425.7, 471.7) | 4464980  (4247816, 4690846) | 222.6  (211.9, 233.6) | -50.1  (-54.1, -46.4) |

**Table S2 The incident cases and age‐standardized incidence of esophageal cancer in 1990 and 2017 and its temporal trends from** 1990 to 2017

|  | 1990 | | 2017 | | 1990-2017 |
| --- | --- | --- | --- | --- | --- |
| Characteristics | **Incident cases**  **No.×10^3^ (95% UI)** | **ASIR per 100 000**  **No. (95% UI)** | **Incident cases**  **No.×10^3^ (95% UI)** | **ASIR per 100 000**  **No. (95% UI)** | **EAPC in ASIR**  **No. (95% CI)** |
| **Overall** | 164.5(157.2,173.9) | 19.4(18.5,20.5) | 234.6(223.2,246) | 12.2(11.6,12.8) | -2.53(-2.90, -2.16) |
| **Sex** |  |  |  |  |  |
| **Male** | 108.1(102.3,114.9) | 26.0(24.6,27.5) | 162.5(152.1,172.9) | 17.4(16.3,18.5) | -1.81 (-2.08,-1.53) |
| **Female** | 56.4(52.7,61.4) | 13.2(12.3,14.4) | 72.2(66.3,78.1) | 7.4(6.8,8) | -2.53 (-2.90, -2.16) |
| **Age** |  |  |  |  | - |
| **0-14** | 0 | 0 | 0 | 0 |  |
| **15 to 19** | 179.8(159.3,205.1) | 0.14(0.13,0.16) | 62.1(53.7,70.9) | 0.08(0.07,0.09) | -2.60 (-2.82 ,-2.38) |
| **20 to 24** | 399.4(362.7,441.5) | 0.30(0.27,0.33) | 116.2(103.7,131.4) | 0.13(0.11,0.14) | -4.45 (-4.95 ,-3.95) |
| **25 to 29** | 633.4(574.3,701.0) | 0.57(0.52,0.64) | 386.6(346.8,429.4) | 0.31(0.28,0.35) | -4.02 (-5.16 ,-2.88) |
| **30 to 34** | 1159.9(1062,1286.3) | 1.31(1.2,1.46) | 770.9(704.5,848.9) | 0.65(0.59,0.71) | -4.32 (-5.27 ,-3.35) |
| **35 to 39** | 2290.3(2079.9,2559.2) | 2.50(2.27,2.8) | 1027.4(935.8,1132.4) | 1.06(0.96,1.16) | -4.18 (-4.66 ,-3.70) |
| **40 to 44** | 5308.0(4843.7,5882.8) | 7.89(7.20,8.75) | 2822.6(2600.1,3070) | 2.55(2.35,2.77) | -5.45 (-5.99 ,-4.92) |
| **45 to 49** | 8086.4(7412.5,8858.4) | 15.64(14.34,17.13) | 8403.5(7818,9061.7) | 6.73(6.26,7.26) | -4.14 (-4.82 ,-3.45) |
| **50 to 54** | 12833.9(11902,13825.4) | 26.85(24.90,28.92) | 15977.1(14892.9,17061.5) | 13.37(12.46,14.28) | -3.07 (-3.76 ,-2.38) |
| **55 to 59** | 18410.4(17225.5,19773.1) | 42.36(39.64,45.50) | 16554.2(15502,17657.7) | 20.55(19.24,21.92) | -2.54 (-3.19 ,-1.88) |
| **60 to 64** | 27595.0(25973.3,29528.1) | 77.85(73.28,83.30) | 37194.2(34832,39507.1) | 44.93(42.07,47.72) | -2.29 (-2.46 ,-2.11) |
| **65 to 69** | 29650.5(27903,31507.3) | 108.34(101.95,115.12) | 44651.7(41591.4,47829.2) | 72.95(67.95,78.14) | -1.77 (-2.08 ,-1.46) |
| **70 to 74** | 27598.6(25966.5,29524.7) | 146.32(137.67,156.53) | 37874.3(34991.9,41152.6) | 92.93(85.86,100.97) | -2.35 (-2.74 ,-1.96) |
| **75 to 79** | 17220.5(16095.3,18565.6) | 150.77(140.92,162.55) | 31169.0(29027.1,33402.3) | 110.36(102.78,118.27) | -1.46 (-1.85 ,-1.06) |
| **80 to 84** | 9087.3(8427.9,9861.9) | 155.43(144.15,168.68) | 22732.5(21229.8,24203.9) | 124.67(116.43,132.74) | -0.83 (-1.19 ,-0.47) |
| **85 to 89** | 3275.2(3025.2,3581.2) | 159.47(147.30,174.37) | 11059.0(10380.6,11781.5) | 132.54(124.41,141.2) | -0.81 (-1.14 ,-0.47) |
| **90 to 94** | 664.0(608.9,731.6) | 157.80(144.71,173.87) | 3277.7(3059.0,3493.0) | 132.52(123.68,141.22) | -0.88 (-1.13 ,-0.64) |
| **95+** | 80.2(72.4,89.3) | 139.88(126.29,155.87) | 544.6(503.8,584.9) | 122.44(113.26,131.50) | -0.57 (-0.81 ,-0.32) |

Abbreviations: ASIR: age‐standardized incidence rate; CI: confidence interval; EAPC: estimated annual percentage change; UI: uncertainty interval.

**Table S3 The number of deaths and age‐standardized mortality rates of esophageal cancer in 1990 and 2017 and the** temporal trends from 1990 to 2017

|  | 1990 | | 2017 | | 1990-2017 |  |
| --- | --- | --- | --- | --- | --- | --- |
| Characteristics | **Deaths**  **No.×10^3^ (95% UI)** | **ASMR per 100 000**  **No. (95% UI)** | **Deaths**  **No.×10^3^ (95% UI)** | **ASMR per 100 000**  **No. (95% UI)** | **EAPC in ASMR**  **No. (95% CI)** | |
| **Overall** | 168.5(160.9,178.2) | 20.5(19.6,21.7) | 212.6(202.7,222.7) | 11.2(10.7,11.8) | -2.59 (-2.92 ,-2.25) | |
| **Sex** |  |  |  |  |  | |
| **Male** | 109.8(103.9,116.5) | 27.5(26.1,29.1) | 154.4(145.0,163.6) | 16.8(15.8,17.8) | -2.15 (-2.46 ,-1.84) | |
| **Female** | 58.6(54.8,64.2) | 14.1(13.2,15.5) | 58.2(53.8,62.6) | 6.1(5.6,6.5) | -3.51 (-3.91 ,-3.1) | |
| **Age** |  |  |  |  |  | |
| **0-14** | 0 | 0 | 0 | 0 |  | |
| **15 to 19** | 144.9(128.7,164.2) | 0.11(0.10,0.13) | 28.6(25.5,32.3) | 0.04(0.03,0.04) | -4.65 (-5.02 ,-4.27) | |
| **20 to 24** | 275.7(251.8,303.5) | 0.21(0.19,0.23) | 47.0(42.9,51.9) | 0.05(0.05,0.06) | -6.47 (-7.08 ,-5.86) | |
| **25 to 29** | 379.1(345.4,418.9) | 0.34(0.31,0.38) | 137.8(127.0,149.9) | 0.11(0.10,0.12) | -5.99 (-7.04 ,-4.93) | |
| **30 to 34** | 772.6(707.9,849.8) | 0.87(0.80,0.96) | 322.8(299.2,348.2) | 0.27(0.25,0.29) | -5.99 (-6.89 ,-5.09) | |
| **35 to 39** | 1726.7(1567.5,1937.4) | 1.89(1.71,2.12) | 494.4(459.0,528.7) | 0.51(0.47,0.54) | -5.77 (-6.39 ,-5.15) | |
| **40 to 44** | 4470.2(4074,4949.3) | 6.65(6.06,7.36) | 1628.2(1514.1,1746.7) | 1.47(1.37,1.58) | -6.80 (-7.45 ,-6.16) | |
| **45 to 49** | 7031.1(6435,7715.6) | 13.60(12.45,14.92) | 5513.0(5162.7,5903.0) | 4.42(4.14,4.73) | -5.22 (-5.94 ,-4.50) | |
| **50 to 54** | 11633.2(10796.1,12549.1) | 24.34(22.59,26.25) | 12228.7(11464.9,13041.5) | 10.23(9.60,10.91) | -3.72 (-4.42 ,-3.02) | |
| **55 to 59** | 17260.4(16175.4,18518.4) | 39.72(37.22,42.61) | 13312.6(12525.1,14152.4) | 16.53(15.55,17.57) | -3.15 (-3.82 ,-2.47) | |
| **60 to 64** | 26848.6(25282.0,28731.7) | 75.75(71.33,81.06) | 31659.0(29655.1,33546.9) | 38.24(35.82,40.52) | -2.81 (-3.01 ,-2.61) | |
| **65 to 69** | 30705.0(28898.6,32615.9) | 112.19(105.59,119.17) | 39796.3(37364.0,42163.9) | 65.02(61.04,68.88) | -2.40 (-2.72 ,-2.09) | |
| **70 to 74** | 29960.5(28220.3,32009.2) | 158.84(149.62,169.7) | 35080.6(32935.5,37526.2) | 86.08(80.81,92.08) | -2.91 (-3.28 ,-2.53) | |
| **75 to 79** | 19968.4(18657.1,21493.3) | 174.83(163.35,188.18) | 30696.8(28916.8,32551.7) | 108.69(102.39,115.26) | -2.10 (-2.52 ,-1.69) | |
| **80 to 84** | 11983.1(11112.6,12979.7) | 204.96(190.07,222.01) | 25212.8(23875.0,26532.9) | 138.28(130.94,145.52) | -1.50 (-1.95 ,-1.06) | |
| **85 to 89** | 4316.0(3987.2,4720.0) | 210.15(194.14,229.82) | 12220.4(11614.3,12857.3) | 146.46(139.19,154.09) | -1.49 (-1.91 ,-1.06) | |
| **90 to 94** | 874.3(801.6,961.7) | 207.76(190.50,228.53) | 3610.7(3408.8,3807.8) | 145.98(137.82,153.95) | -1.56 (-1.90 ,-1.23) | |
| **95+** | 105.5(95.2,117.5) | 184.00(166.13,204.93) | 596.6(556.2,637.5) | 134.13(125.05,143.34) | -1.26 (-1.60 ,-0.93) | |

Abbreviations: ASMR: age‐standardized mortality rate; CI: confidence interval; EAPC: estimated annual percentage change; UI: uncertainty interval.

**Table S4 The number of DALYs and age‐standardized DALY rates of esophageal cancer in 1990 and 2017 and the** temporal trends from 1990 to 2017

|  | 1990 | | 2017 | | 1990-2017 |  |
| --- | --- | --- | --- | --- | --- | --- |
| Characteristics | **DALYs**  **No.×10^3^ (95% UI)** | **age‐standardized DALY rates**  **per 100 000**  **No. (95% UI)** | **DALYs**  **No.×10^3^ (95% UI)** | **age‐standardized DALY rates**  **per 100 000**  **No. (95% UI)** | **EAPC in age‐standardized DALY rates (95% CI)** | |
| **Overall** | 407.8(389.0,431.4) | 446.4(425.7,471.7) | 446.5(424.8,469.1) | 222.6(211.9,233.6) | -3.00 (-3.36 ,-2.65) | |
| **Sex** |  |  |  |  |  | |
| **Male** | 278.3(262.7,296.7) | 609.0(575.0,647.5) | 338.8(317.6,359.4) | 341.2(320.2,361.6) | -2.55 (-2.86 ,-2.24) | |
| **Female** | 129.5(121.1,140.6) | 285.4(266.6,310.8) | 107.7(99.7,116.0) | 107.6(99.7,115.9) | -4.07 (-4.52 ,-3.61) | |
| **Age** |  |  |  |  |  | |
| **0-14** | 0 | 0 | 0 | 0 |  | |
| **15 to 19** | 10261.0(9112.9,11623.5) | 8.08(7.17,9.15) | 2045.4(1817.6,2306.6) | 2.66(2.36,3.00) | -4.62 (-4.99 ,-4.25) | |
| **20 to 24** | 18182.8(16614.2,20004.8) | 13.75(12.57,15.13) | 3118.3(2837.8,3447.7) | 3.40(3.10,3.76) | -6.45 (-7.05 ,-5.84) | |
| **25 to 29** | 23161.6(21093.0,25585.2) | 21.02(19.14,23.22) | 8489.2(7793.0,9256.3) | 6.90(6.34,7.53) | -5.96 (-7.01 ,-4.9) | |
| **30 to 34** | 43388.8(39763.9,47738.4) | 49.10(45.00,54.03) | 18262.2(16940.0,19690.2) | 15.30(14.19,16.50) | -5.97 (-6.87 ,-5.06) | |
| **35 to 39** | 88243.7(80219.4,99171.7) | 96.41(87.64,108.34) | 25449.5(23606.2,27230.6) | 26.14(24.25,27.97) | -5.75 (-6.36 ,-5.14) | |
| **40 to 44** | 206299.1(187835.3,228420.8) | 306.81(279.35,339.71) | 75440.5(70107.5,80897.6) | 68.08(63.27,73.00) | -6.79 (-7.44 ,-6.14) | |
| **45 to 49** | 290565.9(266354.3,318454.5) | 561.96(515.14,615.90) | 228295.6(213669.3,244189.3) | 182.85(171.14,195.58) | -5.22 (-5.93 ,-4.49) | |
| **50 to 54** | 425399.9(394817.0,459409.9) | 890.00(826.02,961.16) | 448734.9(420478.6,478517.7) | 375.56(351.91,400.48) | -3.71 (-4.41 ,-3.01) | |
| **55 to 59** | 548328.4(515089.3,588879.8) | 1261.77(1185.28,1355.08) | 423905.4(399018.2,450394.4) | 526.25(495.36,559.14) | -3.14 (-3.81 ,-2.46) | |
| **60 to 64** | 729097.7(686144.5,779532.0) | 2056.92(1935.74,2199.21) | 859550.4(805598.4,910990.0) | 1038.28(973.11,1100.42) | -2.81 (-3.01 ,-2.61) | |
| **65 to 69** | 694340.5(653877.9,737601.3) | 2537.01(2389.17,2695.08) | 904165.5(849936.5,958542.7) | 1477.16(1388.57,1566.00) | -2.38 (-2.7 ,-2.06) | |
| **70 to 74** | 549462.7(516962,586856.7) | 2913.11(2740.80,3111.36) | 645508.1(606139.7,690977.4) | 1583.85(1487.25,1695.42) | -2.9 (-3.27 ,-2.53) | |
| **75 to 79** | 285506.2(266470.3,307442.9) | 2499.71(2333.04,2691.77) | 438468.5(412968.3,464646.1) | 1552.48(1462.19,1645.17) | -2.11 (-2.53 ,-1.69) | |
| **80 to 84** | 127837.3(118659.6,138559.9) | 2186.55(2029.57,2369.95) | 268807.0(254386.2,282876.2) | 1474.23(1395.14,1551.39) | -1.5 (-1.94 ,-1.06) | |
| **85 to 89** | 33215.9(30741.7,36346.1) | 1617.31(1496.84,1769.72) | 93781.3(89272.7,98743.3) | 1123.93(1069.90,1183.40) | -1.49 (-1.91 ,-1.07) | |
| **90 to 94** | 4642.9(4259.1,5110.9) | 1103.37(1012.17,1214.58) | 19065.7(17968.8,20090.1) | 770.84(726.50,812.26) | -1.58 (-1.91 ,-1.24) | |
| **95+** | 346.7(312.0,387.0) | 604.81(544.40,675.22) | 1892.6(1764.8,2023.8) | 425.50(396.77,455.01) | -1.36 (-1.66 ,-1.06) | |

Abbreviations: CI: confidence interval; EAPC: estimated annual percentage change; UI: uncertainty interval.
